# Host-feeding preference of *Phlebotomus orientalis* (Diptera: Psychodidae) in an endemic focus of visceral leishmaniasis in northern Ethiopia

**DOI:** 10.1186/s13071-015-0883-5

**Published:** 2015-05-13

**Authors:** Araya Gebresilassie, Ibrahim Abbasi, Essayas Aklilu, Solomon Yared, Oscar David Kirstein, Aviad Moncaz, Habte Tekie, Meshesha Balkew, Alon Warburg, Asrat Hailu, Teshome Gebre-Michael

**Affiliations:** Department of Zoological Sciences, Addis Ababa University, Addis Ababa, Ethiopia; Department of Biology, College of Natural Science, Jigjiga University, Jigjiga, Ethiopia; Department of Microbiology and Molecular Genetics, The Institute of Medical Research Israel-Canada The Kuvin Center for the Study of Infectious and Tropical Diseases, Faculty of Medicine, The Hebrew University, Hadassah Medical School, Jerusalem, Israel; Aklilu Lemma Institute of Pathobiology, Addis Ababa University, Addis Ababa, Ethiopia; Department of Microbiology, Immunology and Parasitology, College of Health Sciences, Addis Ababa University, Addis Ababa, Ethiopia

**Keywords:** Bloodmeal, Host preference, *Phlebotomus orientalis*, Tahtay Adiyabo, Visceral leishmaniasis

## Abstract

**Background:**

Blood-feeding behavior studies are important for estimating the efficiency of pathogen transmission and assessing the relative human disease risk. However, in Ethiopia and other parts of East Africa there are large remaining gaps in identifying the feeding habits of *Phlebotomus orientalis*, the vector of *Leishmania donovani*. The aim of the study was to determine the blood feeding patterns of *P. orientalis* in Tahtay Adiyabo district, northern Ethiopia.

**Methods:**

For bloodmeal analysis, sandflies were collected from three different villages of Tahtay Adiyabo district using CDC light traps, sticky traps, and pyrethrum spray catches. Bloodmeal of engorged female sandflies was identified using cytochrome (cyt) *b*-PCR and reverse-line blotting (RLB) and enzyme linked immunosorbent assay (ELISA) assays.

**Results:**

Most (637/641) of the females analyzed were *P. orientalis*. Successful identification of the host from bloodmeals was achieved in 83.03 and 92.1 % using cyt *b* PCR-RLB and ELISA, respectively. Bloodmeal analysis of *P. orientalis* females revealed that they have a range of hosts with predominant preference to bovines followed by donkey, human, goat, sheep, dog, and camel.

**Conclusion:**

Results obtained from bloodmeal analyses demonstrate that the feeding preference of *P. orientalis* is mainly zoophilic, which could vary depending on the availability of hosts.

## Background

Worldwide, leishmaniasis endangers 350 million people in 98 countries and 3 territories, accounting for about 12 million clinical cases each year [1, 2]. This disease is characterized by a spectrum of clinical manifestations, including cutaneous, mucocutaneous and visceral forms. Visceral leishmaniasis (VL), which its causative agent is *L. donovani*, is a common infection and a major public health concern in East African countries with approximately 29,400 to 56,700 new cases each year [2]. In Ethiopia, several active foci of VL are located in southwestern savannah, the southeastern semiarid lowlands, and the Humera-Metema lowlands [3–5]. Recently, new endemic focus has appeared in the semi-arid lowlands of northwestern Tigray Regional State, north Ethiopia, previously considered free from the disease [6].

Transmission of VL occurs when a female sandfly acquires infection feeding on an infected host and transmits the parasite during subsequent feedings after completion of the gonotrophic cycle, during which the parasite takes full development in the gut [7, 8]. In Sudan, South Sudan, northern and southwestern Ethiopia, *P. orientalis* has been indicated as the main vector of VL [9–11].

The natural blood feeding habits of sandfly vectors under natural conditions are determined by examining the bloodmeal origin of field-collected specimens [11–15]. Previously, the host preference of *P. orientalis* in Ethiopia was determined by identification of sources of bloodmeals by counter current immuno-electrophoresis (CCIE) technique [16] and enzyme linked immunosorbent assay (ELISA) [11]. In the Humera-Metema plains, *P. orientalis* exhibits zoophilic behavior by predominantly feeding on bovine blood [10]. Similarly, around Awash Valley (northeast of Ethiopia), bloodmeal analysis of *P. orientalis* revealed that the vector species mainly feeds on domestic animals found in the area [16]. Proper characterization of the feeding habits of sandflies is crucial for developing effective vector control programs. However, no bloodmeal source identification in wild caught *P. orientalis* females was performed in the new VL endemic area of Tahtay Adiyabo district.

Therefore, bloodmeal source identification of wild caught *P. orientalis* in this VL endemic area was conducted using cytochrome *b* PCR (Cyt *b*) and reverse line blotting (RLB) and enzyme-linked immunosorbent assay (ELISA).

## Methods

### Study area

The fieldwork of the study was conducted in three villages of the rural district of Tahtay Adiyabo (14°23'41"N/ 37°46'15"E) in the Tigray Regional State, northern Ethiopia. The topography of the study area is predominantly lowland plain except in the southwest, where it is mountainous. Sheraro, the administrative center of the district, lies 1,028 meters above sea level and has a latitude and longitude of 14°23'41" N /37°46'15" E, respectively. The town is also located about 1,117 km north of Addis Ababa. The three villages were Ademeyti, Lemlem, and Mentebteb. The villages of Ademeyti and Lemlem are approximately 17 and 6 kms northwest and west of Sheraro town, respectively. The third village, Mentebteb is located about 13 km southwest of Sheraro town. The distance between the three villages is about 8–12 km.

The climate is generally sub-tropical-arid, with an extended dry period of nine to ten months. The area has a uni-modal pattern of rainfall (July-September) with a mean annual precipitation of about 600 mm (Ethiopian National Meteorological Agency). March to May is the hottest part of the year with an average temperature of 39 °C at noon and January is the coldest one with an average temperature of 14.2 °C at night.

The villages are situated on hilly outcrops surrounded by large farm fields of vertisol alternating with large tracts of red clay soil. The inhabitants are mainly engaged in the production of cereals and oilseeds and raising different domestic animals such as cattle, sheep, goats, dogs, donkeys, camels and chickens. Furthermore, a wide range of wild animals, including hares, ground squirrels, rodents, reptiles, white-tailed mongoose and foxes are either occasionally or commonly seen.

### Sampling and handling of blood fed females

Females of *P. orientalis* and other *Phlebotomus* spp. (*P. papatasi*, *P. bergeroti* and *P. martini*) with fresh bloodmeals were collected from three different villages of the study area using CDC light traps, sticky traps and pyrethrum spray catch methods between May 2011 and April 2012. Collection of blood-fed specimens from indoors, peri-domestic biotopes (places with human and animal shelters), and agricultural fields on the periphery of settlements was carried out twice a month for three consecutive nights.

Five CDC light traps suspended with 40–50 cm above the ground level were randomly distributed to representative locations of peri-domestic biotopes and agricultural fields. Fifty sticky traps (white A4 sized polypropylene boards coated with sesame oil) were also used for sampling blood-fed females from indoors, peri-domestic and agricultural fields. Moreover, indoor resting sandflies were sampled in the morning (6:00 to 9:00) from ten other randomly selected houses by the application of pyrethrum spray catch method.

The head of each blood-fed female was carefully separated from the thorax and slide-mounted in Hoyer’s medium for species identification using taxonomic keys [17, 18]. The remaining body (thorax and abdomen) were individually placed in empty antibiotic capsules with silica gel grains and cotton pads inside. Likewise, some of blood-fed females were preserved in 70 % ethanol for later DNA extraction. In the laboratory, the specimens were stored at −20 °C for later bloodmeal analysis using ELISA [19] and Cyt *b* PCR and RLB methods [14].

### Bloodmeal determination

Of 824 blood-fed sandflies (820 *P. orientalis*, 1 *P. papatasi*, 1 *P. bergeroti*, and 2 *P. martini*) collected by CDC light traps, sticky traps and pyrethrum spray catches, 641 were randomly selected for bloodmeal identification (Table 1). Selection of sandfly specimens was based on village, capture sites and sampling periods to obtain a representative samples. One hundred and eighty three of those randomly drawn blood-fed samples were analyzed by Cyt *b* PCR-RLB (Table 1). The remaining 458 samples were processed by ELISA method.

**Table 1:** Number of blood fed sandflies tested, listed by species, location, and method

Species	Villages						Total
	Ademeyti		Lemlem		Mentebteb		
	ELSA	RLB	ELSA	RLB	ELSA	RLB	
*P. orientalis*	220	87	230	68	7	25	637
*P. papatasi*	0	0	0	1	0	0	1
*P. bergeroti*	0	0	0	1	0	0	1
*P. martini*	0	1	0	0	1	0	2

### Cytochrome *b* PCR and reverse line blotting

#### DNA extraction

DNA was extracted individually from blood-fed females of sandflies by digestion in a total volume of 200 μL of lysis buffer (50 mM NaCl, 10 mM ethylenediaminetetraacetic acid [EDTA], 50 mM Tris–HCl pH 7.4, 1 % triton X-100, and 200 μg/mL of proteinase K). This was followed by extraction with phenol-chloroform and precipitation using ethanol. The precipitated DNA was suspended in Tris-EDTA (TE, 10 mM Tris–HCl pH 7.4, 1 mM EDTA) buffer at a concentration of 50 μL.

#### PCR amplification of the mtDNA cyt *b* gene

A 344 bp sequence of the conserved region of the mitochondria cyt *b* gene was amplified using bio-tinilated universal primers designed by Abbasi *et al*. (2008). The sequences of the primers used were Cyto1: 5’-CCA TCA AAC ATC TCA GCA TGA TGA AA-3’ (forward primer) and Cyto2: 5’-CCC CTC AGA ATG ATA TTT GTC CTC-3’ (reverse primer). The cyt *b* region was amplified in a total reaction volume of 50 μL consisting of 25 μL Hot start taq Master mix (1.5 mM MgCl_2_, 200 μL each deoxyribonucleotide triphosphates (dNTP) and 75 mM KCl, 10 mM Tris HCl pH8.8) and 0.5 μM of each primer and 5 μL of genomic DNA. The thermo cyclic conditions consisted of 95 °C for 5 min, 35 cycles at 94 °C for 30 s, 55 °C for 30 s, and 72 °C for 1 min; followed by elongation step at 72 °C for 10 min. Cow blood was used as positive control and double distilled water as negative control. The amplified PCR products were used as probes in RLB hybridization reactions followed by chromogenic detection. The methods used by Abbasi *et al*. [14] were followed for immobilization, hybridization, and detection.

#### Species-Specific Probes, Immobilization, Hybridization, and Detection

Species-specific 5'-amino-linked oligonucleotide probes for human, cow, sheep, goat, camel, donkey, dog, mice, brown rat, chickens and a general avian probe developed by Abbasi *et al*. [14] were used in the current study.

The synthetic 5'-end amino modified oligonucleotide probes were covalently linked to nylon membranes through the formation of amide bonds between the carboxyl groups on the nylon and the amino groups linked to the oligonucleotides. Biodyn C (Pall Biomedical, Fajardo, Puerto Rico) nylon membrane were activated in 0.1 N HCl for 5 min, rinsed with DH_2_O and soaked in 10 % 1-ethyl-3-[3-dimethylaminopropyl] carbodiimide (EDC) (Sigma, St. Louis, MO, USA) for 15 minutes. The membranes were rinsed in DH_2_O water and air-dried. Species-specific, 5'-end amino modified oligonucleotides were diluted to 5 p moles/ μL and applied to the membrane in line format using a manifold blotter apparatus.

The nylon membrane sheets with the above mentioned probes were cut at a right angle to the direction of the blot so that each strip contained a section of each probe. Strips were incubated in prehybridization solution (2X SSC [0.15 M NaC1, 0.015 M sodium citrate], 0.1 % sodium dodecyl sulphate [SDS]) for 30 min at 45 °C with gentle shaking. Biotinylated PCR products were denatured by boiling for 5 min and applied to the membrane strips. Hybridization was performed at 46 °C for 1 h followed by a single wash with 0.7X SSC, 0.1 % SDS for 20 minutes. Hybridized biotinylated DNA was detected by incubating the strips in streptavidin-horse radish peroxidase (HRP; diluted in 2X SSC, 0.1 % SDS) for 30 min at room temperature. Strips were washed briefly 3 times in 2X SSC, 0.1 % SDS. For chromogenic detection, a freshly prepared solution containing 0.1 mg/mL of 3,3'5,5' tetramethylbezidine (Sigma), 0.003 % H_2_O_2_ in 0.1 M sodium citrate (pH 5.0) was added. Enhanced chemiluminescent (ECL) detection was performed immediately after streptavidin-HRP incubation and washing steps using EZ-ECL detection kit (Biological Industries, Beit Haemek, Israel).

### Serologic analysis

Bloodmeal origins of freshly fed sandflies were also determined using a direct enzyme (ELISA). The abdomen and thorax of each blood-fed sandfly was individually triturated in 2 ml Eppendorf tubes with micro-tissue grinders to which 50 μl of 0.01 M phosphate buffered saline (PBS), pH 7.2 was added. Samples were then mixed with PBS to desired dilutions and kept in the refrigerator (4 °C) until tested. Sandfly triturate (50 μl) was diluted in PBS (3:50) and 50 μl was added to wells of polyvinyl chloride, U-shaped, 96-well micro titer plates (Dynatech Laboratories, Inc., Alexandria), which were covered and incubated at 4 °C overnight. Plates were washed three times with phosphate-buffered saline, Tween 20, pH 7.2. The plate was blocked using 200 μl of bovine serum albumin/carbonate-bicarbonate buffer (200 mg of bovine serum albumin in 20 ml of carbonate-bicarbonate buffer), and left to incubate for 2 h at room temperature, washed three times with phosphate-buffered saline, Tween 20, pH 7.2. This was followed by the dilution of host-specific peroxidase-conjugated anti-IgG antibodies (anti-human, anti-bovine, anti-donkey, anti-dog, anti-goat and anti-sheep) diluted at 1:2,000; 1:250 1:5000, 1:5000, 1:5000 and 1:5000 in 0.5 % boiled casein containing 0.025 % Tween 20, respectively. Cross-reactions were noted between anti-goat and anti-sheep with the blood samples of cow, human, and donkey and vice versa. Thus, mixed bloodmeals of these antibodies were excluded. The boiled casein was prepared by dissolving 5 g casein in 100 ml of 0.1 N NaOH by boiling, adding 900 ml PBS, adjusting pH to 7.2 adding 0.1 g Thimerosal (Sodium ethylmercurithiosalicylate) and 0.02 gm phenol red. After 1 h incubation, wells were washed three times with 200 μl PBS Tween 20, and then 100 μl of ABTS (2, 2-azino-di- [3-ethyl benzthiazoline sulfonate]) peroxidase substrate (Kirkegaad and Perry Laboratories, Inc.) was added to each well. Negative controls were prepared using unfed laboratory-reared female *P. orientalis* while positive serum controls were done by making host serum: PBS dilutions of 3:50 [12]. Each plate contained a positive control of host species; four negative controls and test samples (1:50 dilution in PBS for all cases). Results were visually assessed, and absorbance was measured with an ELISA reader at 405 nm approximately 30 minutes after addition of substrate solution. Test samples were considered positive if absorbance values exceeded the mean plus three times the standard deviation of four negative controls.

## Results

### Cyt *b* PCR-RLB

A total of 183 blood-fed *Phlebotomus* spp. (180 *P. orientalis*, 1 *P. papatasi*, 1 *P. bergeroti*, and 1 *P. martini*) were analyzed for bloodmeal identification and 168 (91.80 %) were positive to cyt *b* PCR. The remaining 15-bloodmeal samples of *P. orientalis* did not produce distinctive bands for cyt *b* amplifications. All the PCR-positive samples were used for the identification of bloodmeals imbibed in female sandflies using RLB (Table 2). Successful identification of the host from bloodmeals was achieved in 137/165, 1/1, 1/1 and 1/1 of *P. orientalis*, *P. papatasi*, *P. martini* and *P. bergeroti*, respectively. However, some of the samples (i.e., 28) which were positive for cyt *b* PCR amplification did not produce bands (Table 2).

**Table 2:** Bloodmeal sources of *P. orientalis* captured from three different villages and identified using Cyt *b* PCR and RLB

Sources of bloodmeal	Ademeyti	Lemlem	Mentebteb	Total ( %)
Bovine	41 (51.25)	35 (58.33)	16 (64)	92 (55.76)
Human	10 (12.5)	8 (13.33)	4 (16)	22 (13.33)
Goat	3 (3.75)	3 (5)	2 (8)	8 (4.85)
Sheep	1 (1.25)	0	0	1 (0.61)
Camel	0	0	1 (4)	1 (0.61)
Human-Bovine	4 (5)	2 (3.33)	0	7 (4.24)
Human-Bovine-Goat	3 (3.75)	0	0	3 (1.82)
Human-Bovine-Goat-Camel	1 (1.25)	0	0	1 (0.61)
Bovine-Goat	1 (1.25)	1 (1.67)	0	1 (0.61)
Bovine-Sheep	1 (1.25)	0	0	1 (0.61)
Unidentified	15 (18.75)	11 (18.33)	2 (8)	28 (16.97)
Total (+cyt *b* PCR)	80	60	25	165
Negative	7	8	0	15
Total (tested)	87	68	25	180

A high proportion of *P. orientalis* females were found to have fed on bovine blood in all the three study villages (Table 2). In Ademeyti, out of 87 bloodmeals tested 41 (51.25 %), 10 (12.50 %), 3 (3.75 %), 1 (1.25 %) and 10 (12.50 %) contained blood of bovine, human, goat, sheep and mixed hosts, respectively. In Lemlem, 58.30 % and 13.30 % of *P. orientalis* fed on bovine and human blood respectively while the remaining 31.67 % fed on the blood of other hosts. The proportions of *P. orientalis* detected to be positive to bovine, human and goat bloodmeal origins in Mentebteb were 64.00 %, 16.00 %, and 2.00 %, respectively. Besides, large proportions of unidentified bloodmeals were obtained in Ademeyti and Lemlem villages compared to Mentebteb. One specimen of *P. martini* was positive to human blood in Ademeyti. Similarly, *P. papatasi* and *P. bergeroti* each with one specimen contained human blood origin.

**Fig. 1:**
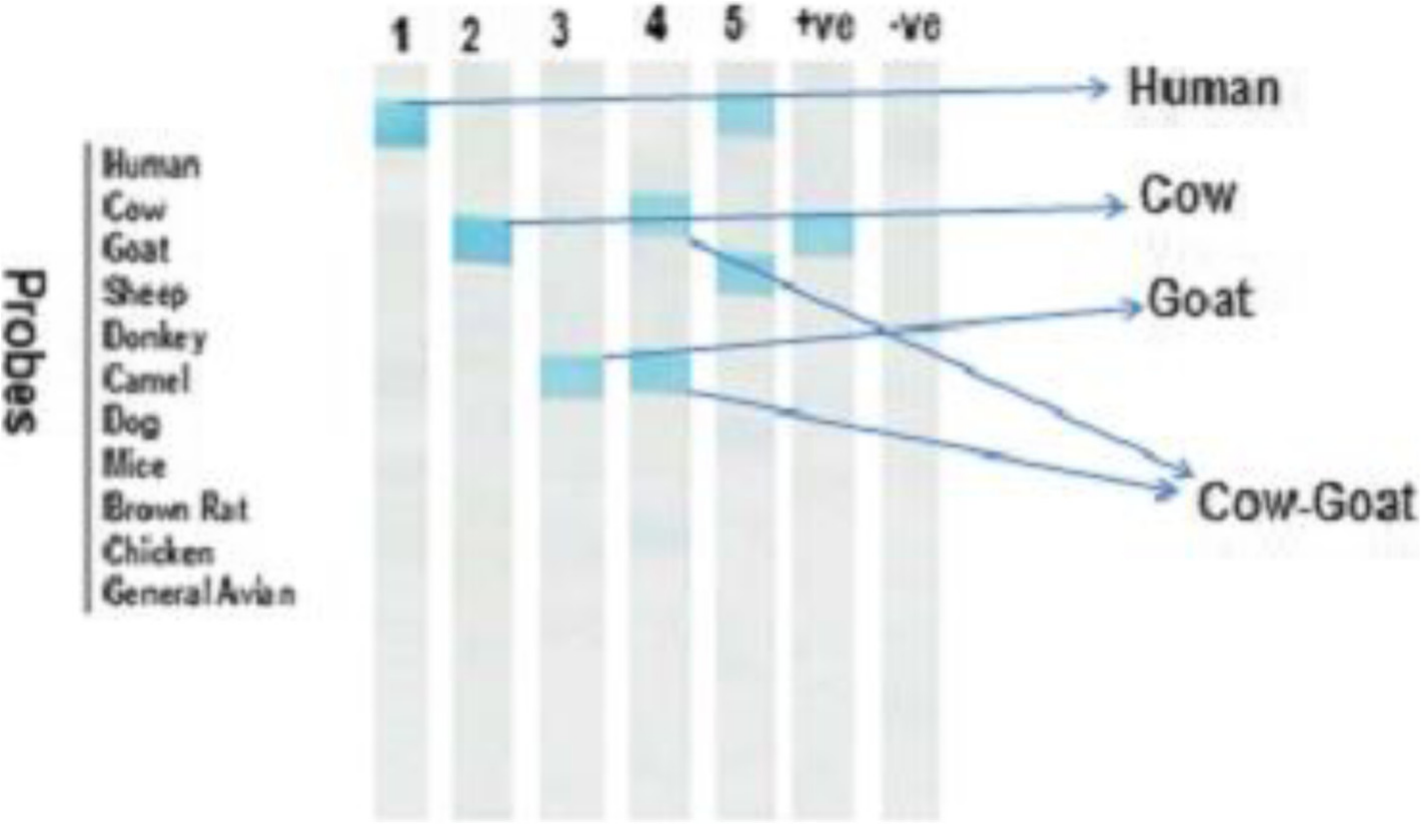
Representative RLB results of Cyt *b* PCR products from blood-fed, *P. orientalis*. Sample number 1: human blood. Sample 2: cow blood. Sample 3: goat blood. Sample 4: cow and goat. Sample 5: human and cow blood. +ve sample is cow blood. -ve sample with no PCR product

In terms of collection habitats, human blood indices for *P. orientalis* were 25.00 %, 6.54 % and 26.32 % indoor, peri-domestic and agricultural fields, respectively (Table 3). For bovines, however, it was 20.00 % indoor, 66.36 % in peri-domestic and 44.74 % in agricultural fields. A single bloodmeal source of sheep and camel was detected in *P. orientalis*. Mixed bloodmeals in *P. orientalis* females, including human-cow, human-cow-goat-camel, human-cow-goat, cow-goat, and cow-sheep were detected using the PCR-RLB technique (Tables 2 and 3; Fig. 1).

**Table 3:** Number and percentage of bloodmeal sources of *P. orientalis* collected from different habitats and detected by Cyt *b* PCR-RLB

Sources of bloodmeal	Indoor	Peri-domestic	Agricultural field
Bovine	4(20)	71(66.36)	17(44.74)
Human	5(25)	7(6.54)	10 (26.32)
Goat	2(10)	1(0.93)	5(13.16)
Sheep	0	0	1(2.63)
Camel	0	0	1(2.63)
Human-Bovine	3(15)	4 (3.74)	0
Human-Bovine-Goat	3(15)	0	0
Human-Bovine-Goat-Camel	1(5)	0	0
Bovine-Goat	0	1(0.93)	0
Bovine-Sheep	0	0	1(2.63)
Unidentified	2(10)	23(21.5)	3(7.89)
Total (+cyt *b* PCR)	20	107	38
Negative	0	12	3
Total (tested)	20	119	41

### ELISA assay

Table 4 shows the bloodmeal origins of *P. orientalis* from different sampling villages. In this assay, 458 blood-fed females of *P. orientalis* (n = 457) and *P. martini* (n = 1) were analyzed, and a general reactivity index of 92.1 % was obtained against antibodies of test animals (human, bovine, donkey, dog, and goat/sheep). Bloodmeal origins from bovine (46.6 %), donkey (9.63 %), human (6.78 %), goat/sheep (5.03 %), and dog (1.75 %) were detected in female *P. orientalis* (Table 4). As well, 103 (22.54 %) of female *P. orientalis* had bloodmeals of mixed origin (Table 4). Hosts for the remaining 8.0 % of the blood samples, could not be identified. One specimen of *P. martini* collected from Mentebteb also had mixed bloodmeal of bovine-donkey-dog.

**Table 4:** Results of ELISA assays on bloodmeals of *P. orientalis* collected from different study villages of Tahtay Adiyabo district

Sources of bloodmeal	Ademeyti	Lemlem	Mentebteb	Total
Bovine	113 (51.36)	98 (42.61)	2 (28.57)	213 (46.61)
Donkey	16 (7.27)	25 (10.87)	3 (42.86)	44 (9.63)
Human	12 (5.45)	17 (7.39)	2 (28.57)	31 (6.78)
Goat/sheep	13 (5.45)	11 (4.78)	0	23 (5.03)
Dog	3 (1.36)	5 (2.17)	0	8 (1.75)
Bovine-Donkey-Dog	10 (4,55	19 (8.26)	0	29 (6.35)
Bovine-Dog	12 (5.45)	8 (3.48)	0	20 (4.38)
Bovine-Donkey	1 (0.45)	0	0	1 (0.22)
Donkey-Dog	4 (1.82)	6 (2.61)	0	10 (2.19)
Human-Bovine-Dog	2 (0.92)	0 (0)	0	2 (0.44)
Human-Bovine	2 (0.92)	7 (3.04)	0	9 (1.97)
Human-Bovine-Donkey	11 (5)	10 (4.35)	0	21 (4.6)
Human-Donkey	0	5 (2.17)	0	5 (1.1)
Human-Dog	1 (0.45)	2 (0.87)	0	3 (0.66)
Human-Donkey-Dog	1 (0.45)	2 (0.87)	0	3 (0.66)
Unidentified	20 (9.09)	15 (6.52)	0	35 (7.66)
Total	220	230	7	457

Further classifications of the bloodmeals by sampling villages and habitat types are presented in Tables 4 and 5. In Ademeyti and Lemlem, the highest proportion of bloodmeal index was for bovine, constituting 51.36 % and 42.61 %, respectively (Table 4). Whereas in Mentebteb the highest, bloodmeal index was for donkey (42.86 %) followed by bovine and human each having 28.57 %.

**Table 5:** Bloodmeal origins of *P. orientalis* collected from indoor, peri-domestic and agricultural field as determined by ELISA assay

Sources of bloodmeal	Indoor	Peri-domestic	Agricultural field
Bovine	8(34.78)	176(51.76)	29(30.85)
Donkey	4(17.39)	34(10)	6(6.38)
Human	6(26.09)	22(6.47)	3(3.19)
Goat/sheep	2(8.7)	8(2.35)	13(13.83)
Dog	0	8(2.35)	0
Bovine-Donkey-Dog	2(8.7)	14(4.12)	13(13.83)
Bovine-Dog	0	18(5.29)	2(2.13)
Bovine-Donkey	0	1(0.29)	0
Donkey-Dog	0	6(1.76)	4(4.25)
Human-Bovine-Dog	0	2(0.59)	0
Human-Bovine	0	8(2.35)	1(1.06)
Human-Bovine-Donkey	1 (4.35)	17(5)	3 (9.57)
Human-Donkey	0	4(1.18)	1(1.06)
Human-Dog	0	2(0.59)	1(1.06)
Human-Donkey-Dog	0	2(0.59)	1(1.06)
Unidentified	0	18(5.29)	17(18.09)
Total	23	340	94

Blood feeding preferences of *P. orientalis* females also varied according to sampling habitats. *P. orientalis* females caught indoor contained bloodmeal origin in the following order: 34.78 %, 26.09 %, 17.39 %, and 8.70 % for bovine, human, donkey, and goat/sheep, respectively (Table 5). In peri-domestic habitat, the highest host preference was for bovine (51.76 %) followed by donkey (10.00 %), human (6.47 %), dog (2.35 %) and goat/sheep (2.35 %). Similarly, the index of bloodmeal for different hosts in agricultural fields ranged from 30.85 % of bovine bloodmeal origin to 3.19 % of human host blood.

## Discussion

In the current study, wild-captured *P. orientalis* females were demonstrated to depend largely on bovine bloodmeals, constituting 55.76 % in PCR-RLB and 46.61 % in ELISA tests. Similar results have been previously recorded for *P. orientalis* in northeast [16] and northwest of Ethiopia [11, 20]. In addition, the role played by cattle as bloodmeal sources for *P. argentipes* was largely indicated in various studies in India [15, 21, 22]. Large proportions of *P. orientalis* contained bloodmeals of bovine, which could be related to their availability and abundance as well greater release of kairomones, compared to other animal hosts in the area [23, 24]. Secondly, this could also be associated with capturing of most engorged *P. orientalis* females from peri-domestic biotopes. In our study area, cattle are raised in large numbers by villagers and are usually kept in enclosures close to residential houses. The accessibility of bovine blood hosts to questing *P. orientalis* females in the peri-domestic habitat may provide zooprophylactic barrier potentially reducing human-vector contact, or it may aggravate the risk of VL infection. Thus, the putative role of cattle in the epidemiology of VL requires further validation.

Less proportion (9.6 %) of donkey bloodmeal origin was detected in the bloodmeal analysis. However, a recent host choice study in the same area showed that *P. orientalis* females are attracted and engorged avidly on donkey [25]. Discrepancy between the two methods could be attributed to the lesser abundance of donkeys in the study villages, thereby reducing their accessibility for sandfly bite. The role of donkeys in the epidemiology of VL is a subject of further study.

Importantly, 8.50 % (in both assays) of the wild-caught specimens of *P. orientalis* females contained human blood origin. This finding supports the likelihood that *P. orientalis* is the vector of VL in these parts of East Africa since human biting by a sandfly vector is a minimum requirement for disease transmission [26]. Furthermore, bloodmeal source from goat, sheep, dog, and camel was detected, showing the evidence of being opportunistic feeder with higher degree of zoophily on a broad range of host species.

The apparent presence of multiple bloodmeals from a single specimen in some females is a strong evidence of the eclectic diet of *P. orientalis*. This behavior is a common phenomenon in sandflies and therefore it may be a result of the difficulties sandflies face in freely engorging on a single host due to host defensive mechanisms, little or no exposed host skin or the difficulty to locate adequate skin blood capillaries [13]. For instance, *P. orientalis* females analyzed for bloodmeal in northeast Ethiopia had 54.30 % multiple bloodmeals from available vertebrate hosts [18], the predominant being cattle-camel hosts (60.00 %). This host-feeding behavior can influence pathogen transmission through increased frequency of vector-human contact, or possibly reduce vector-human contact if some bloodmeals are taken from alternative mammalian hosts.

Results of PCR-RLB assay were comparable with ELISA in the detection sensitivity of bloodmeal hosts in field-collected females of *P. orientalis* in this study (91.80 % versus 92.20 %). However, PCR-RLB method is known to possess the unique ability to analyze bloodmeal from the blood-fed sandflies having minute quantities of DNA (>0.1 pg), it is a more rapid technique and can easily differentiate between host species in mixed bloodmeals sources in a single insect [14, 15]. Furthermore, combining bloodmeal analysis with parasite detection in one Multiplex PCR-based RLB hybridization assay can be used for identifying the vectorial role of sandfly species and the reservoir status of various mammalian hosts [27]. Therefore, all these better qualities of PCR-RLB in comparison with ELISA are useful in studying bloodmeal sources in vectors of zoonotic diseases in general and leishmaniasis in particular for vector incrimination and reservoir host determination.

Prominently, 17.0 % in PCR-RLB and 7.7 % in ELISA assays of the bloodmeal samples were not from any of the eleven oligonucleotide probes or antibodies tested. These unidentified bloodmeals could belong to different species of wild animals found in the area. Alternatively, either degradation of residual host DNA [28] or the very small quantities of bloodmeals in case of ELISA [29] could be the possible reasons for the reduced detection success.

## Conclusions

Our results demonstrated that the feeding preference of *P. orientalis* is primarily zoophilic, which possibly vary depending on the accessibility of bloodmeal hosts. Furthermore, the higher predilection of *P. orientalis* to bite cattle in the peridomiciliary could be exploited for killing sandfly vectors using insecticide treated animals. As sandflies ingest the blood from treated animals, they will ingest the blood with insecticide and adult sandflies die before oviposition, leading to suppression of sandfly population. Reductions in density of sandfly vectors around human dwellings represent a decrease in human-vector contact, which could play a crucial role in minimizing the exposure of people to *L. donovani*.
